# Successful completion of clinical electives – Identification of significant factors of influence on self-organized learning during clinical electives with student focus groups

**DOI:** 10.3205/zma001185

**Published:** 2018-08-15

**Authors:** Natalie Rausch, Sigrid Harendza

**Affiliations:** 1 Universitätsklinikum Hamburg-Eppendorf, III. Medizinische Klinik, Hamburg, Germany

**Keywords:** clinical elective, medical studies, practical skills, self-organized learning

## Abstract

**Background: **The Medical Licensure Act prescribes a total of four months of clinical electives in which the medical students are to work in a self-organized manner in outpatient and inpatient care. Since no specific learning objectives or learning content are given and students come into contact with different structures of outpatient and inpatient care, the learning success in a clinical elective is often rather random. In order to make self-organized learning (SOL) in clinical electives as effective as possible, we identified factors in the area of inpatient care that have an influence on SOL and thus the learning success during a clinical elective.

**Methods: **To investigate this question a qualitative and explorative approach was chosen. In 2015, a total of 21 students from semester 1 to 11 participated in six semi-structured focus group discussions at Hamburg Medical Faculty. In these, the students were asked about their experiences and expectations with regard to SOL in clinical electives. The interviews were transcribed literally and analyzed using Grounded Theory in parallel to further data collection.

**Results: **Three main categories were identified, which had an impact on SOL in clinical electives, each with two sub-categories: People (elective students and physicians), learning itself (learning content and learning process) and the framework (local conditions and organizational structure). For example, elective students exhibiting openness and self-initiative as well as a good working atmosphere and few hierarchical structures were conducive to SOL, while shyness and lack of integration into the ward’s medical team inhibited SOL. A mentor formally assigned to the student can promote SOL through guidance, supervision and the transfer of responsibility. Continuous feedback from mentors or peers promotes SOL. Framework conditions, such as a smooth administrative organization, also affect SOL, but elective students have limited influence over these.

**Conclusion:** The creation of suitable framework conditions and considering the needs of the people involved in clinical electives and the requirements of learning itself are necessary steps in order to enable successful SOL during clinical electives. Suitable framework conditions could be compiled and widely disseminated on an empirical basis. Training for teachers and elective students on various aspects of clinical electives, from professional behavior to practical skills, could be a suitable preparatory measure to promote SOL in clinical electives and contribute to a better learning success of the elective students.

## Zusammenfassung

**Hintergrund: **Die Ärztliche Approbationsordnung schreibt insgesamt vier Monate Famulatur vor, in welchen die Medizinstudierenden selbstorganisiert in der ambulanten und stationären Krankenversorgung mitarbeiten sollen. Da keinerlei konkrete Lernziele oder Lerninhalte vorgegeben sind und die Studierenden mit unterschiedlichen Strukturen der ambulanten und stationären Krankenversorgung in Kontakt kommen, ist der Lernerfolg in einer Famulatur oft eher zufällig. Um selbstorganisiertes Lernen (SOL) in Famulaturen möglichst effektiv zu gestalten, ermittelten wir Faktoren im Bereich der stationären Versorgung, die einen Einfluss auf das SOL und damit den Lernerfolg einer Famulatur haben.

**Methoden:** Zur Untersuchung dieser Fragestellung wurde ein qualitativer, explorativer Ansatz gewählt. Im Jahr 2015 nahmen an der Medizinischen Fakultät Hamburg insgesamt 21 Studierende der Semester 1 bis 11 an sechs semistrukturierten Fokusgruppengesprächen teil. In diesen wurden die Studierenden zu ihren Erlebnissen und Erwartungen im Hinblick auf SOL in Famulaturen befragt. Die Interviews wurden wörtlich transkribiert und parallel zur weiteren Datenerhebung mittels Grounded Theory ausgewertet.

**Ergebnisse:** Es wurden drei Hauptkategorien mit jeweils zwei Subkategorien identifiziert, die Einfluss auf SOL in Famulaturen hatten: Personen (Famulierende und Ärzte), das Lernen selbst (Lerninhalte und Lernprozess) und die Rahmenbedingungen der Famulatur (örtliche Gegebenheiten und Organisationsstruktur). Beispielsweise zeigten sich ein von Offenheit und Eigeninitiative geprägtes Verhalten der Famulierenden sowie eine gute Arbeitsatmosphäre und niedrige Hierarchien förderlich für SOL, während sich Schüchternheit und fehlende Integration in das ärztliche Stationsteam hemmend auf SOL auswirken. Ein den Studierenden fest zugewiesener Mentor kann durch Anleitung, Supervision und Übergabe von Verantwortung das SOL fördern. Kontinuierliches Feedback durch Mentoren oder Peers fördert das SOL. Rahmenbedingungen, wie beispielsweise eine reibungslose administrative Organisation, beeinflussen ebenfalls das SOL, können jedoch wenig durch die Famulierenden beeinflusst werden.

**Schlussfolgerung: **Die Schaffung geeigneter Rahmenbedingungen und die Berücksichtigung der Bedürfnisse der im Rahmen einer Famulatur beteiligten Personen und der Erfordernisse des Lernens selbst sind notwendig um erfolgreiches SOL in Famulaturen zu ermöglichen. Geeignete Rahmenbedingungen könnten auf empirischer Basis zusammengestellt und allgemein verbreitet werden. Ein Training für Lehrende und Famulierende zu diversen Aspekten einer Famulatur, vom professionellen Verhalten bis zu praktischen Fertigkeiten, könnte eine geeignete vorbereitende Maßnahme zur Förderung von SOL in Famulaturen sein und zu einem besseren Lernerfolg der Famulierenden beitragen.

## Introduction

The content and structure of the study of medicine in Germany is regulated on the one hand by the Medical Licensure Act [https://www.gesetze-im-internet.de/_appro_2002/BJNR240500002.html, accessed: 29.12.2017] and on the other hand by the study regulations of the universities, which prescribe content and study hours for each subject. According to the Medical Licensure Act, there are structural specifications for type and duration of clinical electives [https://www.gesetze-im-internet.de/_appro_2002/BJNR240500002.html, accessed: 29.12.2017]. However, in terms of content and choice of disciplines, no special requirements are made, so the learning success of clinical electives depends essentially on the SOL of the students and thus becomes random. Whether such unstructured clinical electives achieve the desired effect of familiarizing students with the provision of care in medical facilities https://www.gesetze-im-internet.de/_appro_2002/BJNR240500002.html, accessed: 29.12.2017] is also questioned in the international literature [1], [2]. At the start of their training, 60% of the residents feel that they are not well prepared for their work [3] and several studies show that residents have deficits in examination techniques and the diagnostic process [4], [5], [6]. Therefore, efforts are made to provide structure to the content of clinical electives [7] or to enrich them with structured courses, for example on physical examination skills [8].

It is even more important that students learn SOL, which includes above all self-esteem, academic commitment and time management as essential factors [9], as this is essential for the future medical practice [10] and could also be practiced in clinical electives and thus improve student learning success. SOL in a self-organized learning environment [11], which is essentially what a clinical elective represents, i.e. a learning environment without defined teachers and learning objectives, includes aspects of self-directed (SDL) as well as self-regulated (SRL) learning [12]. For self-directed learning to take place effectively, students must be able to set their own goals and to achieve these through self-regulated learning [12]. However, who learns well in self-regulated way is not necessarily also good at self-directed learning [12], because in medical studies most learning objectives are dictated by the curriculum and the setting of self-imposed goals is usually not practiced. It is well known that at the start of their studies, medical students have a high motivation for SOL, which decreases sharply after the first year of study [13], [14]. It is also known that the way medical students perceive their learning environment is associated with whether and how they use SOL [15]. In order to optimize learning success of elective students, it is important to know the factors that influence SOL, SDL and SRL. Only when these factors are known will teachers be able to best support and assist elective students. Therefore, the aim of this qualitative study was to gather data from students on factors, which have a positive or negative impact on SOL in clinical electives and to develop a proposal on how to optimize learning success in clinical electives based on these criteria.

## Methods

A semi-structured discussion guide was developed to identify aspects of SOL in clinical electives which, based on a SWOT analysis [16], was structured around four major areas: Strengths of a clinical elective, weaknesses of a clinical elective, possibilities for improvement and dangers of structuring clinical electives. Further detailed questions were assigned to these four areas, guided to some extent by the results of studies that examined expectations and experiences in specific phases of medical studies [17], [18], [19], but left room for follow-up questions if necessary. The guiding idea was to ask students who had not yet completed a clinical elective about their hopes, expectations and fears about their first clinical elective, while students who had already completed at least one clinical elective were to be given the opportunity in the interviews to report on their positive and negative experiences in the context of their clinical elective and to make suggestions for improvement. The complete discussion guide can be found in the Attachment 1. In addition, the following socio-demographic data of the participating students were collected anonymously: age, gender, semester, as well as the number and disciplines of the completed clinical electives.

In 2015, students from the 1^st^, 3^rd^ and 5^th^ semesters of medicine, who studied at Hamburg Medical Faculty, were invited to the focus group discussions by email after a lecture for their respective semester. Participation was voluntary and anonymous. A declaration of no objections to this study by a member of the ethics committee of the Hamburg Medical Association regarding the anonymized focus group investigations amongst students is available. The discussions were held separately for each semester. At the end of each focus group discussion, a book voucher for €25 was awarded through a raffle. All discussions were moderated by NR. The discussions were audio recorded and transcribed verbatim. The evaluation was done using Grounded Theory [20]. Independent of each other the authors inductively formed codes and categorized them. In joint comparison, the codes were discussed and refined or rejected. In the course of data collection it turned out that it might be interesting to have conversations with students in even higher semesters or with those who have already progressed to their Practice Year, because on the one hand they would already have completed all clinical electives and on the other hand would be able to consider and judge SOL in clinical electives from a different context. Therefore, students from the 9^th^ and 11^th^ semesters were invited to participate. Due to the higher number of volunteers from the 11^th^ semester, two focus group discussions took place with this cohort. These discussions were also conducted by NR. After the analysis of the sixth discussion (in total), the codes were found to be saturated. In the next step, the identified codes were grouped into categories and content super-categories. Afterwards, those coded passages were selected from each category, which best illustrated the corresponding category.

## Results

A total of six focus group interviews were conducted with a total of 21 students (eight male, 13 female). The average age of all participants was 25.2±3.9 years, 28.6% of all participants had not yet completed any clinical elective, 33.3% had completed at least one and 38.1% had completed all required clinical electives. Among the students who had already completed at least one clinical elective at the time of this study, the most frequently mentioned disciplines were internal medicine (9), general practice (8) and radiology (7)

**Figure 1:**
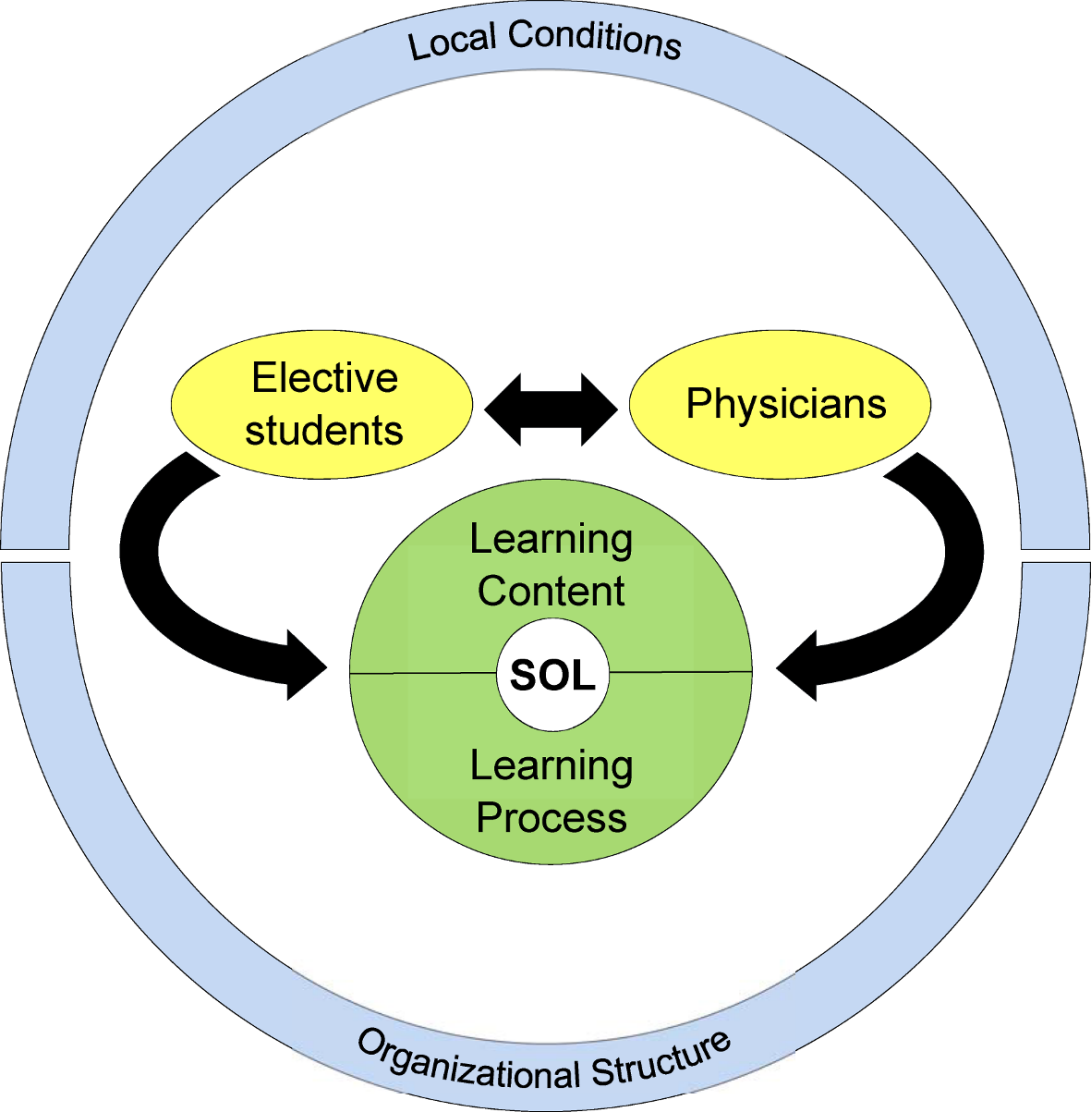
Main categories (yellow: People, green: Learning, blue: Framework) with their sub-categories and influences (arrows). SOL: self-organized learning

The analysis of the focus group discussions revealed three main categories which influenced SOL, each with two essential subcategories, and which are shown in Figure 1 (Fig. 1): People (elective students and physicians), learning (learning content and learning process) and environment of the clinical elective (local conditions and organizational structure). There were reciprocal influences between elective students and physicians, which in each case influenced the learning content, the learning process and thus self-organized learning. The local conditions as well as the organizational structure represent the external framework of the clinical elective. Their various sub-aspects showed influences on the elective students and physicians as well as on the learning content and the learning process, but are little influenced themselves in turn.

The motivation of the elective students, their behavior as well as their self-assessment in terms of knowledge and skills were identified as crucial factors influencing SOL (see Table 1 (Tab. 1)). Positive motivation of the elective students has a positive effect on the motivation experienced by the physicians and the learning process, which encourages SOL. Through their own behavior elective students can decisively influence the learning success or failure of their clinical elective, with both personality characteristics and behavioral norms constituting important influencing factors. Behavior driven by self-initiative and openness has a positive effect on the learning process, while insecurity or shyness, for example out of consideration for the busy residents, tend to inhibit the learning process and SOL. Passive behavior is also used by the elective students in a targeted way to avoid tasks regarded as unpleasant (for example, taking blood), which may hinder SOL if these skills have not yet been mastered. In terms of behavioral norms, punctuality, politeness and a well-groomed appearance were factors that were considered important by the elective students in order to convey a positive image to the physicians and nurses, thereby increasing their motivation to teach and to increase the learning success of the elective student. Another sub-category deemed to have an impact on successful SOL amongst elective students was self-assessment with regard to a realistic appraisal and communication of their own abilities and knowledge as well as their limitations.

Regarding the physicians, three sub-categories were identified which had an impact on the elective students’ SOL: the motivation of the physicians, their expectations of the elective students and their role as mentors (see Table 2 (Tab. 2)). Physicians perceived by elective students to be motivated increased elective students’ SOL and thus the learning success of a clinical elective. The motivation to teach is attributed by the students to the workload of physicians, personality traits of physicians and their position in the hospital hierarchy. Students would like the physicians to express their expectations towards the elective students so they can organize their SOL and are not inhibited by uncertainty in their learning process. Often the attending physicians are also unclear about what the duties of an elective student are in general, something which can hinder the learning process. The elective students would like to have a mentoring physician who supports their SOL during the clinical elective and enjoys teaching. They consider younger physicians to be more suitable for such a task and see the workload of physicians as an obstacle to successful mentoring. Elective students expect mentors to serve as role models, provide guidance, and assign tasks appropriate to the elective students’ skill level in a responsible way to foster the elective students’ SOL individually.

In terms of learning content, seven sub-categories were identified (see Table 3 (Tab. 3)). It was most frequently mentioned that the elective students want to experience the work environment and patients’ progression and set themselves no concrete learning objectives in terms of SOL. Students also consider it to be important to learn basic practical skills and to practice these in a self-structured manner, as well as basic nursing skills, which are apparently not or not sufficiently learned during the nursing internship, but are usually presupposed in clinical electives. The students also expect to be introduced to the process of clinical decision-making and to learn professional behavior during a clinical elective in order to practice these in SOL. Five sub-categories were identified for the learning process (see Table 4 (Tab. 4)). It is important that an induction period is planned for the start of clinical electives in order to get into the process of SOL. Learning itself is seen as a student’s duty, with targeted thematic preparation, continuous practice and taking on responsibilities seen as important aspects for encouraging SOL. Mentoring by supervisors as well as peers (e.g. students in their Practice Year) and continuous feedback were important to students in order to be able to structure their SOL well during the clinical elective.

In terms of the framework conditions of a clinical elective, the local conditions with the size of the hospital and the working atmosphere were mentioned as important sub-categories (see Table 5 (Tab. 5)). The size of the hospital is, depending on the personal learning preferences, evaluated differently in regard to the range of subjects and tasks. Integration into the medical and nursing team is also seen as an essential factor for successful SOL. Organizational structures prior to as well as during a clinical elective play a role for the impact of a clinical elective on SOL (see Table 6 (Tab. 6)). Preparations before a clinical elective could include structured introductions into skills and supervised practice which may prove beneficial for SOL. A voluntary preparatory course for duties such as ward rounds and writing discharge letters could reduce elective students’ uncertainty and increase their self-confidence for SOL during the clinical elective. Uncertainties regarding the process and content of a clinical elective as well as the selection of a suitable place can have negative effects on SOL. Amongst the organizational factors that can inhibit or encourage SOL during a clinical elective were administrative aspects, introductory events, permanent medical supervisors who accompany the learning process, and a clinical elective guide for supervisors and elective students were mentioned.

## Discussion

It is known from a study of SOL in the clinical environment that students find it very difficult to deal with SOL when there is a lack of support and guidance [21]. The three main categories identified in our study that impact SOL in clinical electives - people, learning, and framework conditions - along with their sub-categories provide good starting points for encouraging SOL in the clinical environment. According to our analysis, the motivation of the students plays an essential role for SOL in clinical electives. This can be strengthened with the help of a preparatory course that familiarizes students with their role in a clinical elective [22]. In addition, students’ motivation also depends on being integrated into the medical team [23] and students who are more proactive get more opportunities to practice and take on more responsibilities, while shyness or insecurity inhibit SOL [24]. Integration into the medical team and demonstrating self-initiative should also be part of preparatory courses. Learning with peers, other elective students or students in their Practice Year, which some elective students take up independently and which offers good support in the clinical environment with busy physicians [25] and encourages SOL through integration into ward procedures [26] and greater autonomy over learning content [27], could also be the subject of targeted interventions before the first clinical elective. Residents were identified more strongly as key contacts by the elective students in our study rather than consultants. It was mentioned that residents untrained in the guidance of elective students often step in as supervisors [28], which has a negative impact on the interaction of students and supervisors and thus on the students’ SOL; also, trainings of residents in emotional intelligence are helpful in encouraging self-initiative and thus SOL of elective students [29]. Likewise, the mentoring by physicians desired by students during clinical electives is beneficial for SOL and does not seem to pose an additional burden on the residents [30].

In addition to experiencing the work environment, which is not a learning objective in the narrow sense of the term, the participants of our study had concrete wishes for SOL with respect to basic practical and nursing skills, professional behavior, guided experience of pathological findings and practicing clinical decision-making. For example, learning outcomes which include professional interaction and good inter-disciplinary communication could be developed for clinical electives [31], which could be practiced both in advance and practically during clinical electives. Similarly, specific tasks should be developed which offer opportunities for developing SOL and taking on responsibilities, as research has shown that students who had been integrated into teams and given responsibilities self-assessed their skills in physical examination, clinical decision-making, and developing treatment plans as significantly better [32]. The setting of personal learning goals in the learning process is an important prerequisite for effective SOL in clinical electives [33]. It makes sense to provide guidance to students herewith before their first clinical elective or as part of the clinical elective [34]. However, as it is also known that students often are reluctant to communicate their learning goals to mentors and to ask them to carry out or take over certain activities [35], appropriate behavior in this regard should also be practiced prior to the first clinical elective. Guidance and feedback are also essential features of a successful learning process [36] and lead to good learning outcomes, especially through peer-teaching [37]. Reasons for this include explanation of issues in comprehensible language [38] and a learning atmosphere that enhances self-confidence [39] and in which students dared to ask questions [26], [38]. Elective students should be prepared to seek such encouraging learning situations themselves. In addition, training for students in their Practicel Year can demonstrate how they can effectively integrate elective students into ward procedures and patient care [40]. Teaching simultaneously promotes their own self-confidence, clinical skills and communication skills [41].

Prior to their first clinical elective there are various aspects of local conditions and organizational structures relating to the framework conditions that the elective students should investigate in order to optimally design their SOL. A university hospital offers elective students chances to acquaint themselves with rarer diseases and complex cases in specialist departments [42], while smaller hospitals offer more frequent patient contact and more opportunities for acquiring a routine in clinical decision-making [43] with greater satisfaction with the learning environment [44]. This could be explained in a preparatory event so that students can plan their SOL more effectively. Doing the first clinical elective in internal medicine also leads to better academic performance during subsequent clinical electives [45], as training in internal medicine provides a good basis for medical knowledge in other specialties [46]. Such evidence-based findings on clinical electives should also be made available to students for planning their clinical electives. In relation to organizational structures within hospitals, some elective students still receive poor access to electronic health records for successful SOL [47], although more than 90% of deans of education considered students’ documentation should be an important component of learning [48]. If students are given the opportunity to document their own findings and processes, i.e. are actively involved in patient treatment, this promotes further reflection and structured thinking [49]. The fact that a survey of students’ notes in 82% of cases revealed findings which had been fabricated or had been arrived at through incorrect examination techniques by students [50] underlines the desire of elective students for medical supervision and feedback on elementary clinical skills [51] to enable better SOL. Hospitals organizing electives should consider these aspects. A prospectively controlled study was able to show that supervised involvement of students in the diagnostic process can even contribute to improved numbers of correct diagnoses [52].

A strength of this study lies in the survey of students ranging from those having completed all clinical electives to those who have yet to begin a clinical elective, which means that both qualitative aspects of the expectations regarding SOL as well as aspects of good and bad experiences with SOL in clinical electives fed into the analysis. A weakness of this study is that only students from one university participated. A second limiting point of the study is that only the student’s view of SOL in clinical electives was recorded. For a more comprehensive picture, including the views of supervisors on students’ SOL in clinical electives could have provided additional insights. Despite these limitations, due to the extensive data collection, important indications for suitable organization of SOL in clinical electives can be derived. For clinical electives in outpatient care, identifying specific aspects which encourage SOL could similarly be determined and provide interesting starting points for improving learning outcomes.

## Conclusions

In order to facilitate successful SOL in clinical electives, the creation of suitable framework conditions and consideration of the needs of the people involved as well as suitable learning processes are required. Suitable framework conditions that encourage SOL could be summarized in a brochure based on the discussed empirical findings and made available electronically to students and hospitals. For supervisors and elective students, training courses could be offered that deal with aspects of professional behavior, fundamentals of learning, practical skills, and taking responsibility. Through such trainings, all people involved in clinical electives could be informed about appropriate means which encourage SOL during clinical electives and could contribute to maximizing learning success of elective students through their successful integration into ward procedures. 

## Acknowledgements

We thank all the medical students who participated in the focus groups.

## Competing interests

The authors declare that they have no competing interests.

Parts of this article are taken from the PhD thesis of NR and were presented by her in a an oral presentation at a conference of the Society for Medical Education in Bern in September 2016.

## Supplementary Material

Discussion guide for 1st and 3rd semester

## Figures and Tables

**Table 1:**
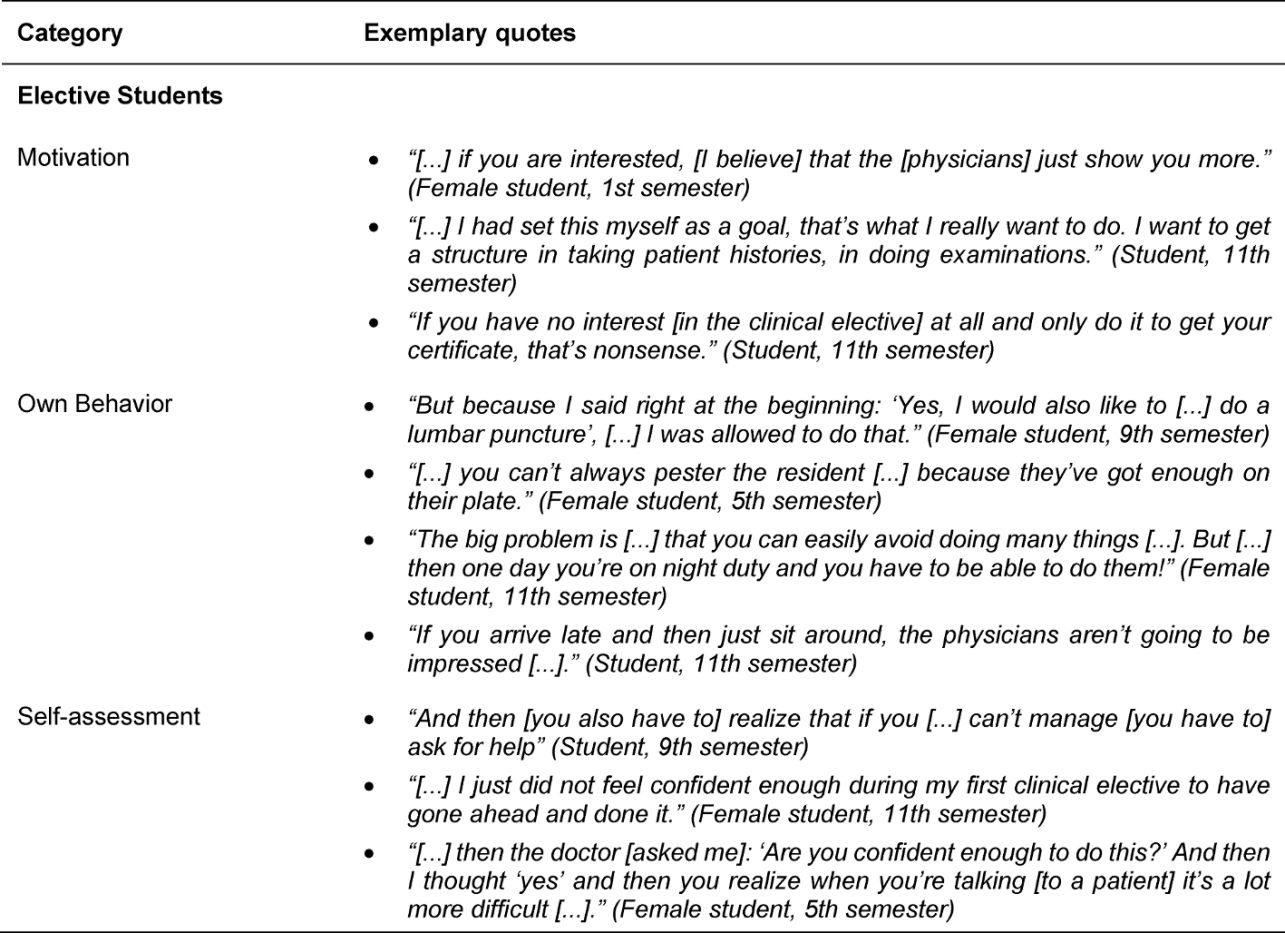
Exemplary quotes for the main category People/Elective Students

**Table 2:**
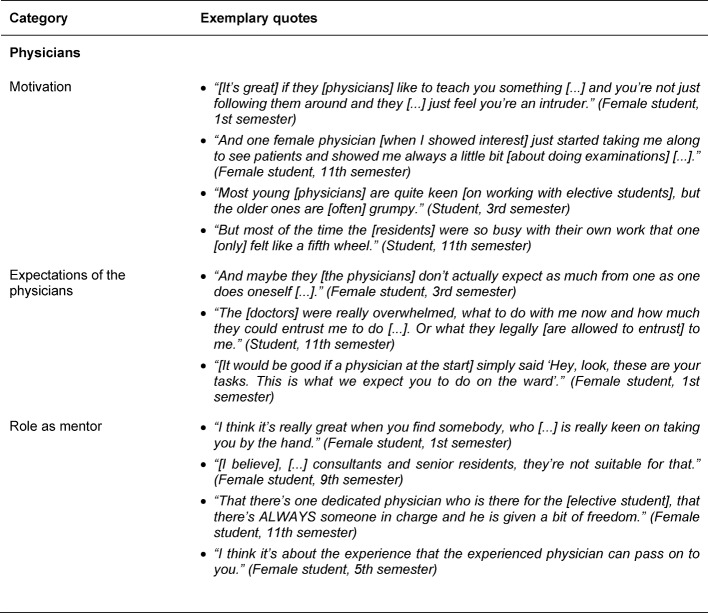
Exemplary quotes for the main category People/Physicians

**Table 3:**
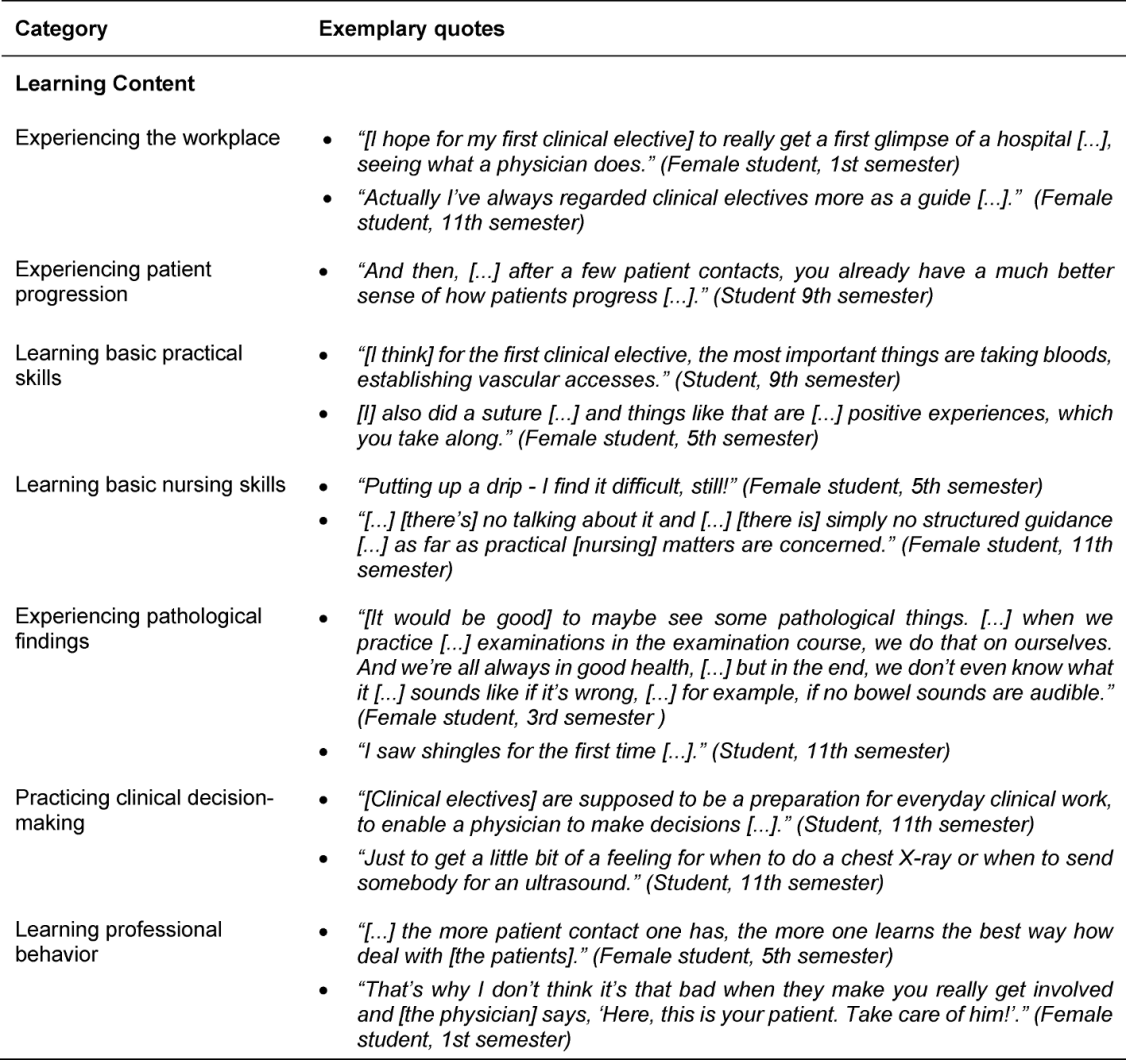
Exemplary quotes for the main category Learning/Learning Content

**Table 4:**
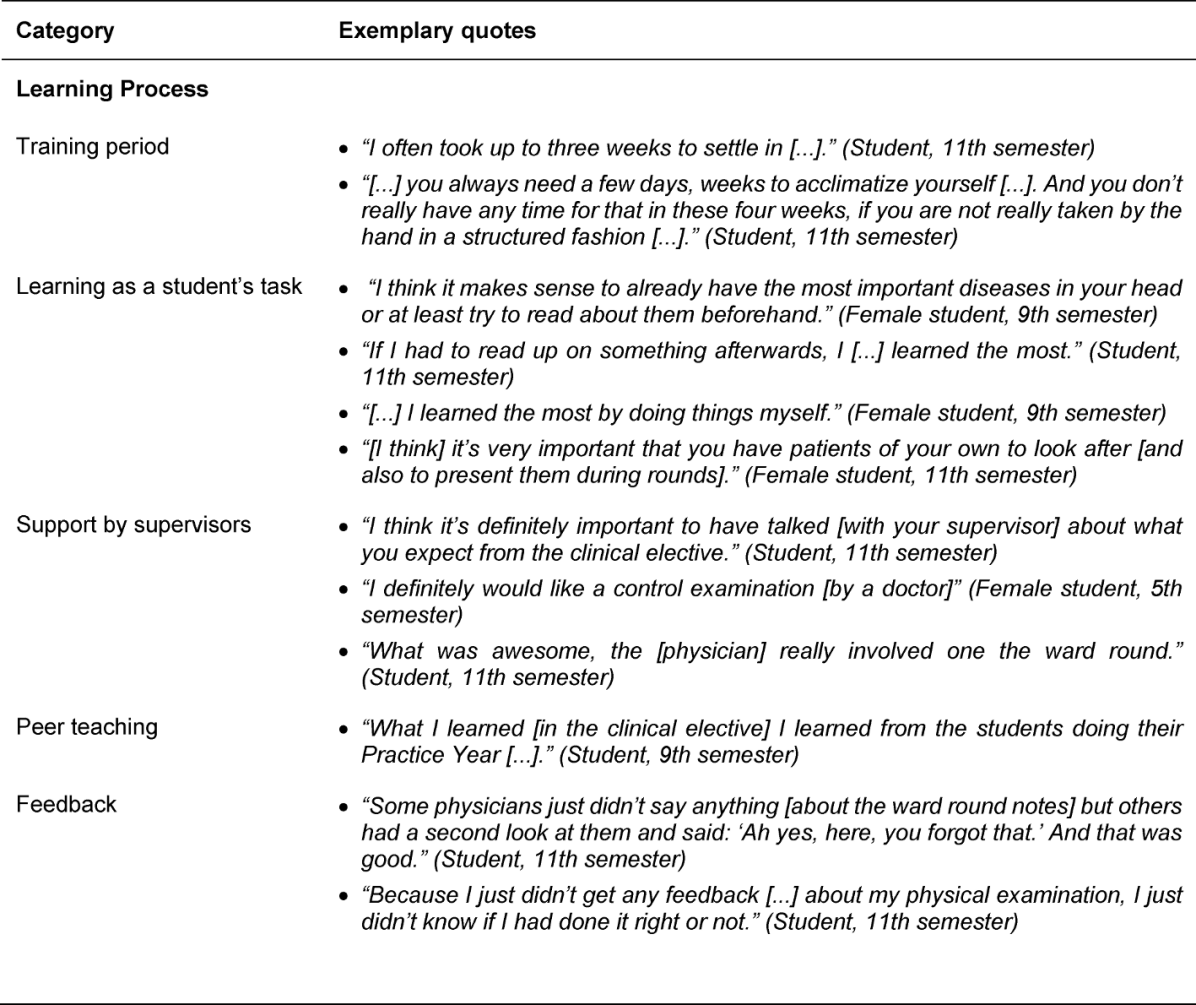
Exemplary quotes for the main category Learning/Learning Process

**Table 5:**
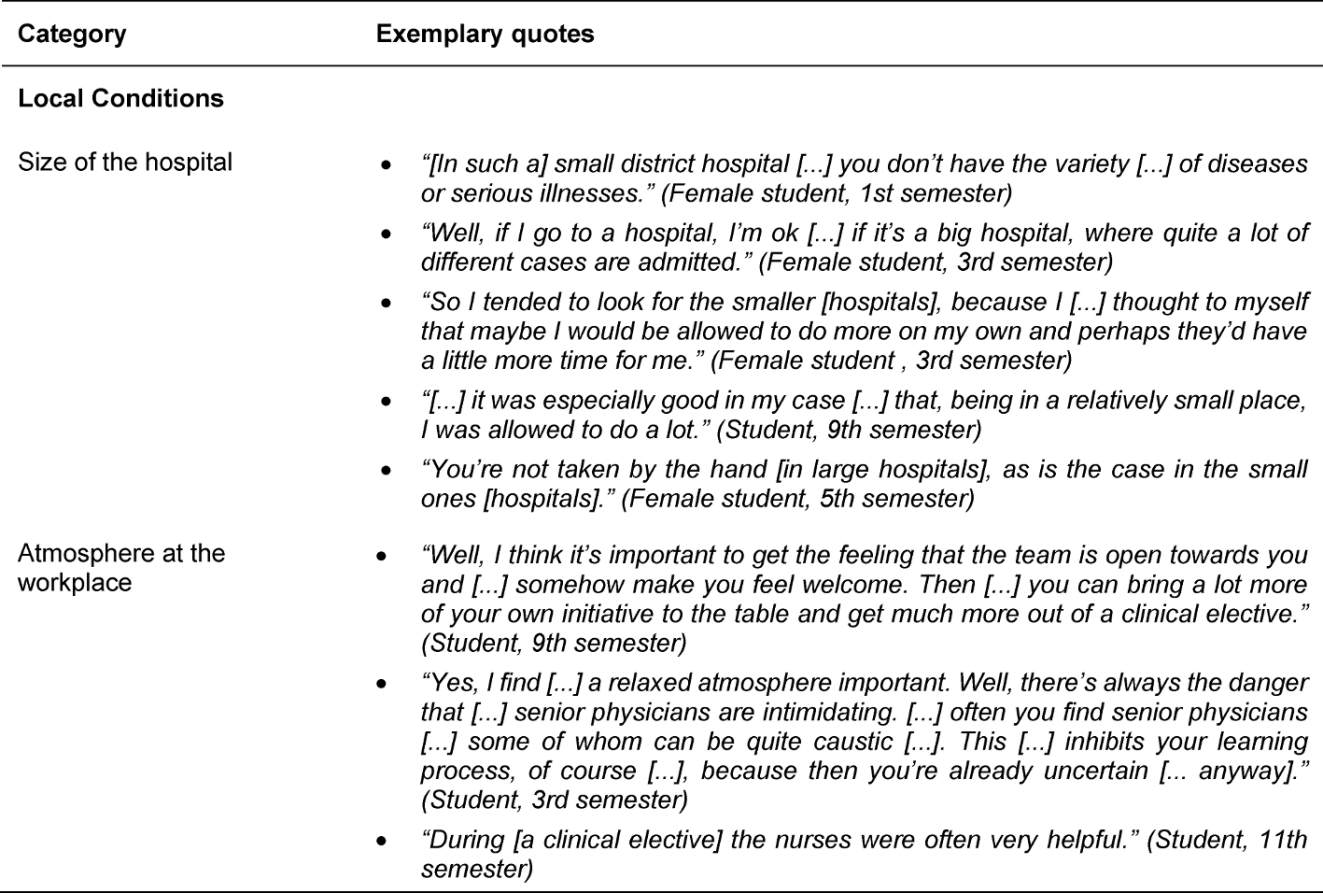
Exemplary quotes for the main category Framework/Local Conditions

**Table 6:**
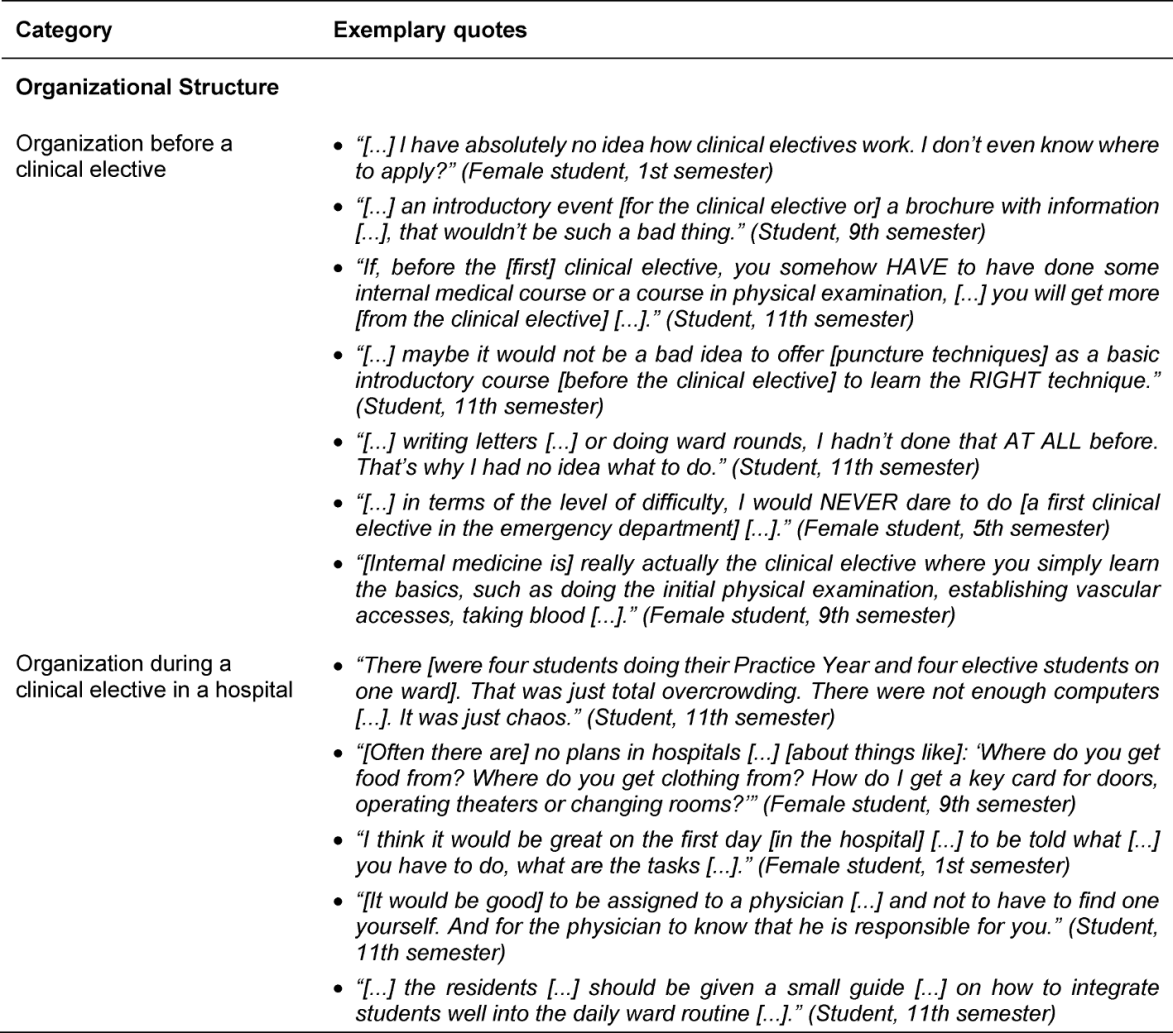
Exemplary quotes for the main category Framework/Organizational Structure
