# Supplementary material for: Successful completion of clinical electives – Identification of significant factors of influence on self-organized learning during clinical electives with student focus groups
Source: GMS J Med Educ. 2018 Aug 15;35(3):Doc39. doi: 10.3205/zma001185 (PMC6120151; doi:10.3205/zma001185)
Supplement: Discussion guide for 1st and 3rd semester [file JME-35-39-s-001.pdf]

Discussion guide for 1st and 3rd semester

What do you imagine to be especially good in a clinical elective? (STRENGTHS)

- What criteria do you use to select a place for your first clinical elective?
- What do you wish to learn in your first clinical elective?
- Which practical skills would you like to learn or to perform?
- What do you wish to have mastered by the end of your first clinical elective by all means?
- In what way will you learn the most in a clinical elective?

What do you imagine to be not so good in a clinical elective? (WEAKNESSES)

- Is there anything you are particularly afraid of in your first elective?
- What could lead to disappointment with your clinical elective?
- How would you feel, if you got the impression on your ward that your presence annoys? How would you behave in such a case?
- In case you make bad experiences during your first clinical elective, e.g. a situation like the one just described, what would this mean for your further clinical electives?
- What would make you quit a clinical elective prematurely?

How could a clinical elective be improved or, respectively, its learning effectiveness?

- What could you do yourselves to improve clinical electives?
  - Would you prepare for a clinical elective? If yes, how?
  - Should a clinical elective be debriefed? If yes, why? And with whom best?
- How could a medical faculty / the curriculum foster good learning success of a clinical elective?
  - Should there be opportunities to prepare for a clinical elective? If yes, what kind?
  - Should there be a guideline for electives? If yes, what should be in it?
- What could the respective ward / the physicians / the nurses / the Practice Year students offer to make the experience of a clinical elective good?
  - Should an elective student have a constant contact person/mentor? Who should this be (Practice Year student, resident, consultant)?
  - What should the supervision by a mentor look like?
  - Should there be little quiz or feedback rounds on the ward?
  - How and by whom should learning objectives be defined?
  - Should there be feedback or an evaluation at the end of a clinical elective?

If there was a preparation for clinical electives, what would it look like and what could be problematic with it?

Discussion guide for 5th to 11th semester

What was particularly good during your clinical electives? (STRENGTH)

- Which criteria did you use to select your clinical elective(s)?
- How was your work on the ward organized? Did you feel welcome? Was there an introduction?
- How was the supervision?
- What were your learning objectives for the first clinical elective? Were these fulfilled?
- Which practical skill did you learn or did you execute?
- What were the patient contacts or the patient care like?
- Were there difficult / straining situations? How were they dealt with?
- Was there a particularly good event that you will not forget?
- How did you learn the most?
- *(only 11th semester: did you feel well prepared for the Practice Year by your clinical electives? If not, what would have had to be been different in the clinical electives?*

What was not so good during your electives? (WEAKNESSES)

- What leads mostly to disappointments during a clinical elective according to your opinion?
- How did negative experiences during your first clinical elective have an influence on you?
- Did you cease a clinical elective prematurely or what would need to happen for you to do so?

How could a clinical elective be improved or its learning effect be increased, respectively?

- How could you improve clinical electives yourselves?
  - Is it important to prepare for a clinical elective? If yes, how and by what means?
  - Would a reflection after a clinical elective be important? If yes, how and with whom should the reflection take place?
- How could a medical faculty / the curriculum foster good learning success of a clinical elective?
  - Should there be learning opportunities to prepare for a clinical elective? If yes, what kind?
  - What should a guideline to accompany a clinical elective contain?
  - Should there be feedback in the following semester?
- What could the respective ward / the physicians do for improvement?
  - Should an elective student have a constant contact person/mentor? Who should this be (Practice Year student, resident, consultant)?
  - What should the supervision by a mentor look like?

- Should there be little quiz or feedback rounds on the ward?
- How and by whom should learning objectives be defined?
- Should there be feedback or an evaluation at the end of a clinical elective?

- **If there was a preparation for clinical electives, what could be problematic with it?**
